# More Christmas Festivities at the Hospitals

**Published:** 1887-01-15

**Authors:** 


### More Christmas Festivities at the Hospitals.

By our Special Commissioner.

Much that is pleasant.

While outside London has been frost-bitten and ice-bound, or, what has been more intensely uncomfortable, reeking in a slow thaw, and ankle-deep in snow and slush, it has been very pleasant this Christmas time to turn into the Hospital wards. Here at least are cleanliness and warmth, a bright, cheery aspect, and a genial atmosphere of practical benevolence in striking contrast with the dull, depressing appearance and the rough turmoil of the streets. And it is not only the material cheer and comfort manifest in these places; but it distinctly raises one's appreciation of average human nature to see so many of the officials and friends of the hospitals ready and eager to co-operate for the benefit of the inmates. "Here they are," they seem to say, "from tenderest infancy to hoary age, every one with his own special affliction, and all of them separated from friends and families at a time when most people like to be at home, if they have any home. Let us see what we can do to brighten then up a little and give them some little share in the festivity of the season." In all sorts of ways, this desire has been manifesting itself this Christmas, and though the thought must now and again cross the mind of the on-looker upon some of the entertainments, that among the occupants of the beds of hospital wards, there must be a good many who secretly rather dread the influx of hundreds of visitors who come to scrutinise and interrogate very often with scarcely as much delicacy as could be wished ; there can be little doubt that by the vast majority of patients well enough to bear it, the little outburst of gaiety at Christmas time is highly appreciated. But, apart from public or semi-public entertainments, there has been going on in the hospitals this Christmas much that it is very pleasant to think of.

THE BROMPTON CONSUMPTION HOSPITAL.

At the Brompton, for instance, Christmas Day was ushered in by the sisters and nurses perambulating the wards in procession, singing carols ; and, as the procession would include several with good musical education and well-trained voices, the effect, as may be imagined, was very pleasing. The day before, the Bishop of London had held a confirmation in the hospital chapel, and after the carol-singing on Christmas morning, what is described as a bright and cheerful eleven o'clock service was held. Then came the Christmas feast, which one good lady supplemented with a case of oranges, and another with a supply of tobacco for those who were smokers, and presents for those who were not. In the evening, music, singing, and games were engaged in for the benefit of such of the 321 patients in the hospital as could be present; the popular Lady Superintendent, Miss Abbott, and the equally popular Dr. Waugh, the Resident Medical Officer, having taken a prominent part in the proceedings. On the Tuesday following, Christmas carols and sacred solos were given in the entertainment room by a trained choir; and on Thursday, the same prettily decorated chamber was brilliantly adorned by an immense Christmas tree provided by the Misses Heddy who have made a similar present in several previous years. It is interesting to observe that the donors of the gifts with which this tree was enriched, included not only many private individuals whose creeds and characters no doubt were diversified enough, but also the Editor of *Truth*, and the secretaries of the Religious Tract Society. It is the abstract religious notions that divide the world. When we come to put our abstract notions into a concrete and practical form, we can most of us work harmoniously enough. Half the creeds of Christendom may chirp in the branches of one Christmas tree, and there will be no quarrelling so long as they will be content to express themselves in a real ministry instead of barren wrangling. The distribution of the gifts from the tree was under the supervision of Father Christmas himself, cleverly impersonated by Mr. F. C. Evill, one of the resident medical staff, whose identity was effectually concealed in the bluff, hearty, jovial exterior supposed to be appropriate to the presiding genius of the season. The whole institution, it may be added, was tastefully decorated, and a happy time seems to have been spent by all, thanks to the officials and nurses, and to the kindly co-operation of the veteran secretary, Mr. Henry Dobbin.

THE LONDON HOSPITAL.

At the London they had a grand day for the children on Tuesday, the 4th of January. This institution is so large that the infants' share in it is commonly regarded as only a small one. Yet, they had in their cots here in the Victoria wards alone last year, no less than 600 children. The two "Victoria" wards, devoted to accident and surgical cases, constitute in themselves quite a large children's hospital, and exceedingly pretty they looked last week with their blazing fires, their brilliant flags and glittering evergreens, and the sweet little faces of the children—many of them so pleasant and happy in the midst of it all. Few of the visitors who moved about the wards last Tuesday week, could very clearly have realized what many of these children were in their indigenous dirt and rags—their unkempt, neglected, repulsive filthiness. As soon as a child is brought in here, every article of clothing is stripped from it, and it is well bathed and clad in the hospital garb ; the old trappings—often mere filthy rags—being bundled back to the child's home.

**Something like a transformation.**

The transformation is often a surprising one. From the rags and dirt and foul neglect of the gutter child often is revealed the little cherub, that a Rubens might paint or a princess adopt for her own. As these little ones sit here in their snowy white cots and their crimson flannels and play with their smart little toys, one forgets really that many of them have come in here literally from the gutters. Usually, they take very readily to the warm bath, and evidently enjoy it. Now and again, however, they get one to whom the outer crust seems to have become indispensable to comfort. "What are you crying for?" a child was asked here the other day. "I wants to go 'ome, I do," was the tearful answer. "What do you want to go home for?" "'Cause I don't like bein' washed, I don't. They never washes me at 'ome." A very significant peep into "home" life, this. Another, even more striking, was afforded by the mother of a tiny patient here, whom the nurses, somehow, could not get to lie properly in bed. The little thing apparently could not be comfortable lying straight in the middle of the bed but betrayed an odd disposition to wriggle into the corners, and lie crosswise in all sorts of awkward positions. "How is it she does not seem to care to lie in bed?" the mother was asked one day. "Well, you see, m'am," replied the mother, "she ain't used to it. She goes out with me in the truck, and she mostly sleeps in one corner of it at 'ome." The little mite, it seemed, had been so used to snuggling down in the truck, that a soft bed with plenty of room in it was a luxury she did not understand and could not appreciate.

**A Happy Party.**

There were about 130 children in the two wards on Tuesday week, and about twenty more were brought from the other parts of the hospital. Some of the children at the London are distributed about the adult wards, and there is a children's medical ward, in addition to those devoted to surgical and accident cases. From this medical ward, however, not many were well enough to be brought. A few were transferred for the occasion, and the whole of the children were brought together in the middle of the floors to give them the best chance of seeing the entertainments provided for them; the cots being laced together by red braid to keep visitors out from among them. Mr. Robert Barclay had given two very fine trees, and one of them, laden with all sorts of shining knicknacks and toys, was placed in each ward, where they were lighted by members of the Hospital Committee, to the intense delight of the little party, many of the members of which had been even more interested and excited in the preparations of the previous day or two than in the actual festival. From two till three o'clock, there was an entertainment of conjuring in one ward, and in the other a performance of Punch and Judy. It was after this that the trees were illuminated, and the distribution of gifts was made, and at four o'clock the friends of the children were admitted to see them and their treasures; the invited guests—very numerous considering the wretched weather through which they had to make their way—having by this time been conducted over the new home for nurses which has added so much to the comfort and efficiency of this great establishment. There are some 200 nurses engaged in this hospital, and this home affords them comfortable accommodation for meals and for sleeping. They have here also a pleasant sitting-room as well as a sick-room, to which any of the nurses who may become disabled for duty may retire and be themselves the subjects of medical treatment. Entertainment Day at the children's wards of the London Hospital appears to have passed off very satisfactorily to the children, and must in many ways have been interesting to the visitors.

### THE EVELINA HOSPITAL FOR CHILDREN.

A Harbour of Refuge.

And the same may be said of the admirable institution in the Southwark Bridge Road, the Evelina Hospital for Sick Children. To anyone who has ever inspected this establishment, it is not surprising to be told that by the poor people around, both the old and the young, it is regarded with quite an affectionate interest. "Dear Miss Cross," wrote the mistress of a neighbouring Board School, some little time ago, "will your Committee kindly accept the enclosed sovereign as a donation to the Evelina Hospital? It has been brought to me by my poor girls in farthings and halfpence; and, I know, represents a heap of self-sacrifice. 'Evelina' is a name endeared to them all, and is a very harbour of refuge in their days of sorrow."

Fete Day.

Last Friday, the 7th of January, was their fête day here, and though the day was wet and the streets deplorably muddy, they had a gathering of visitors almost greater than could be conveniently taken in. Two large wards and two small ones were very elaborately decorated, together with the corridors and staircases. This hospital stands exactly opposite to the head-quarters of the Metropolitan Fire Brigade. Captain Shaw is a Member of the Committee, and on these occasions one or two of his men are always at hand to help with flags and festoons. Between the firemen and the nurses and medical officers, a really very showy display of greenery and colour was made. Chinese lanterns in pretty groups were suspended from trails of foilage stretched on lines drawn "taut" to and fro across the wards, as only professional hands could draw them, and the effect when all was lighted up was very pretty—perhaps a little too subdued and wanting in brilliancy, but this only brought out with brighter effect the glitter of the great Christmas trees, one in each of the four wards, like all the rest heavily laden with treasures gathered together in almost overwhelming abundance from all sources. The wards of the Evelina are themselves very bright and cheerful; and last Friday with their flowers and evergreens, their flags and lamps, their pictures and illuminated texts and mottoes, their toys and their brightly lighted Christmas trees, they were particularly cheerful and gay. On the staircase; and in each of the two smaller wards, a musical-box kept up a succession of lively airs, while in each of the large wards a piano was provided, and some really excellent music was performed by visitors or by the officers of the hospital.

Wanted, Practical Sympathy.

This is a very gratifying list of kindly doings, and though the cynic may have his little snarl at Christmas sentimentality, even he, one would think, would be unable to find much fault with a little festivity for afflicted children who are equal to anything of the kind. But neither the cynic nor his snarl is of much importance any way. The world is the better for this sort of thing, and if all who have been present at any of these gatherings, and have been touched by the wee faces, and the pitiable afflictions of the children, will only bear them in mind, and make some little personal effort and sacrifice for the welfare of the institutions that have to bear the labour and the expense of them; then, not only will these Christmas festivals have been pleasant, but they will have done real and substantial good. Pray, good people, do not let all your kindly emotions bubble away in vapour. Bottle them up a little and convert them into a real force for helping on the great machinery of our hospital charities.

